# Workmen's Compensation

**Published:** 1911-09-23

**Authors:** R. Langton Cole

**Affiliations:** 23 Throgmorton Street, E.C.


### WORKMEN'S COMPENSATION.

*To the Editor of* The Hospital.

Sir,—In reference to the Scotch "frost-bite" case reported in your issue of the 26th ult., I think you have overlooked the English case of a baker's assistant, decided a few months ago, in which the Court held that the employer was not liable. The cases were very similar, and the decisions seem to be contradictory.—Yours truly,

23 Throgmorton Street, E.C., R. Langton Cole.

September 14.

